# The Effect of Salt on the Gelling Properties and Protein Phosphorylation of Surimi-Crabmeat Mixed Gels

**DOI:** 10.3390/gels8010010

**Published:** 2021-12-23

**Authors:** Yajun Zhu, Yufeng Lu, Tao Ye, Shaotong Jiang, Lin Lin, Jianfeng Lu

**Affiliations:** 1 Engineering Research Center of Bio-Process, Ministry of Education, Hefei University of Technology, Hefei 230009, China; zyjdyx2@mail.hfut.edu.cn (Y.Z.); lyfyyds@mail.hfut.edu.cn (Y.L.); yetao430@mail.hfut.edu.cn (T.Y.); jiangshaotong@hfut.edu.cn (S.J.); 2 Key Laboratory for Agricultural Products Processing of Anhui Province, Hefei University of Technology, Hefei 230009, China; 3 School of Food and Biological Engineering, Hefei University of Technology, Hefei 230009, China

**Keywords:** surimi, crabmeat, NaCl, gelling properties, protein phosphorylation

## Abstract

The effects of different salt additions (1.0%, 1.5%, 2.0%, 2.5%, 3.0%, and 3.5%) on the gelling properties and protein phosphorylation of the mixed gels (MG) formed by silver carp (*Hypophthalmichthys molitrix*) surimi with 10% crabmeat were investigated. The MG’s breaking force, deformation, gel strength, and water-holding capacity (WHC) increased as the salt concentration increased. The intrinsic fluorescence intensity of the samples initially decreased and then increased, reaching the lowest when the NaCl concentration was 2.5%. The result of SDS–polyacrylamide gel electrophoresis indicated that large aggregates were formed by protein–protein interaction in the MG containing 2.5% or 3.0% NaCl, decreasing the protein band intensity. It was also found that with the addition of NaCl, the phosphorus content initially increased and then decreased, reaching the maximum when the NaCl concentration was 2% or 2.5%, which was similar to the changing trend of actin band intensity reported in the results of Western blot. These results revealed that the amount of salt used had a significant effect on the degree of phosphorylation of the MG protein. The increase in phosphorylation was linked to improved gelling properties, which could lead to new ideas for manufacturing low-salt surimi products in the future.

## 1. Introduction

Surimi is a Japanese word and a term for concentrating myofibrillar protein, which is derived from the deossified material produced in fish that has been continuously washed and mixed with cryoprotectants [1]. Silver carp (*Hypophthalmichthys molitrix*) is a common freshwater fish species in China, and its low cost makes it popular for commercial production. Although the production of this freshwater fish is high, its price remains modest as compared with that of the other freshwater fish species [2,3]. Therefore, to increase the product’s added value, silver carp meat was processed into surimi products as low-fat and high-protein processed food. Surimi food, such as fish balls and crabsticks, is popular with consumers as a type of ready-to-eat food because of its nutrition, taste, and convenience [4]. In general, after the addition of salt, surimi is chopped and heat-treated to form elastic gel products through the formation of protein networks by promoting covalent and non-covalent interactions [5]. Besides enhancing the flavor of surimi products, salt is used to extract myofibrillar protein during surimi processing to obtain the desired texture and taste. Without the addition of salt, it would not be possible for myosin heavy chain (MHC) to aggregate due to the lack of myosin solubilization and unfolding [6]. Furthermore, the decrease in salt concentration adversely affects the extractability and solubility of surimi proteins, resulting in a poor gel structure and mechanical properties [7]. High salt intake, according to the World Health Organization (WHO), has negative effects on blood pressure, thereby increasing the risk of cardiovascular diseases [8].

Furthermore, as society has progressed, more and more consumers have become increasingly conscious of eating healthily. Consequently, the surimi industry must match that pace and work toward reducing salt in processed foods [1]. However, it might be a great challenge since salt plays a vital role in surimi gelation. Recently, researchers have been focusing on producing low-salt surimi (less than 2.5% (W/W) NaCl) products, which have the same quality as normal-salt surimi products, either through processing techniques or salt substitution strategies. High hydrostatic pressure (HHP) treatment has a certain stabilizing effect on the protein structure of surimi gels with different NaCl additions (0.3% and 3.0%), according to Cando, Herranz, Borderías, and Moreno [9], and 300 MPa HHP treatment could significantly increase the gel properties of a low-salt (0.3%) sample. Moreover, given the right processing circumstances, lipids [10], proteins [11], and polysaccharides [12] improve the surimi gel texture under appropriate processing conditions. Although the development of healthy surimi products has made some progress, the mechanism of salt affecting the properties of surimi products has not been thoroughly studied; thus, more research is needed in this area.

Chinese mitten crab (*Eriocheir sinensis*), also known as river crab, is an important aquatic economic animal in China. The crabmeat contains abundant proteins, essential amino acids, long-chain omega-3 fatty acids, vitamins, and minerals and offers a unique flavor profile [13]. Crabs that have reached sexual maturation in the first year rather than the second year of their life are usually called precocious crabs. Their nutritional value is similar to normal crabs, and in some respects, even higher [14,15]. However, the market value of precocious crabs is relatively low because of their small size; this presents a challenge in river crab aquaculture. Adding precocious crabmeat to surimi products can not only improve the nutritional value and flavor of products but can also increase the added value of precocious crab and reduce the economic losses of fishermen [16]. Therefore, further research is needed to determine the processing feasibility and action mechanisms of mixed gels formed by surimi and precocious crabmeat.

Protein phosphorylation is one of the most important methods for controlling protein activity and function. Some researchers have summarized that phosphorylation can affect the protein structure at the local and overall level, and phosphorylation can also serve as an enzyme recognition site rather than producing structural changes to catalyze changes in protein conformation [17]. Chemical phosphorylation, enzyme phosphorylation, glucose-6-phosphate-binding phosphorylation, and dry-heating phosphorylation have recently been used to study the phosphorylation modification of dietary proteins. Phosphorylation can alter the functional properties of dietary proteins such as solubility, thermal stability, emulsifying properties, foaming properties, and gel properties [18]. The phosphorylation of myofibrillar proteins (MP) can regulate muscle contraction, thereby affecting the dissociation of actomyosin. Chen et al. [19] discovered that myofibrillar protein phosphorylation may affect meat rigor mortis via contractile machinery and glycolysis and ultimately impact the meat tenderness. A study has shown that salting reduces the phosphorylation of MHC, myosin-binding protein C (MyBPC), and actin (AC). Thus, the meat tenderness is improved [20].

The purposeful alteration of proteins to improve their properties has garnered a lot of attention lately. Although salt is an important component in surimi gel production, its impact on muscle protein phosphorylation is unclear. In this study, the effects of salt addition on the properties and protein phosphorylation of silver carp surimi and crabmeat mixed gels were investigated, to elucidate the action mechanism of the salt’s effect on surimi products’ quality from the perspective of phosphorylation to provide new ideas for future production of high-quality surimi products.

## 2. Results and Discussion

### 2.1. Breaking Force and Deformation

**Figure 1:**
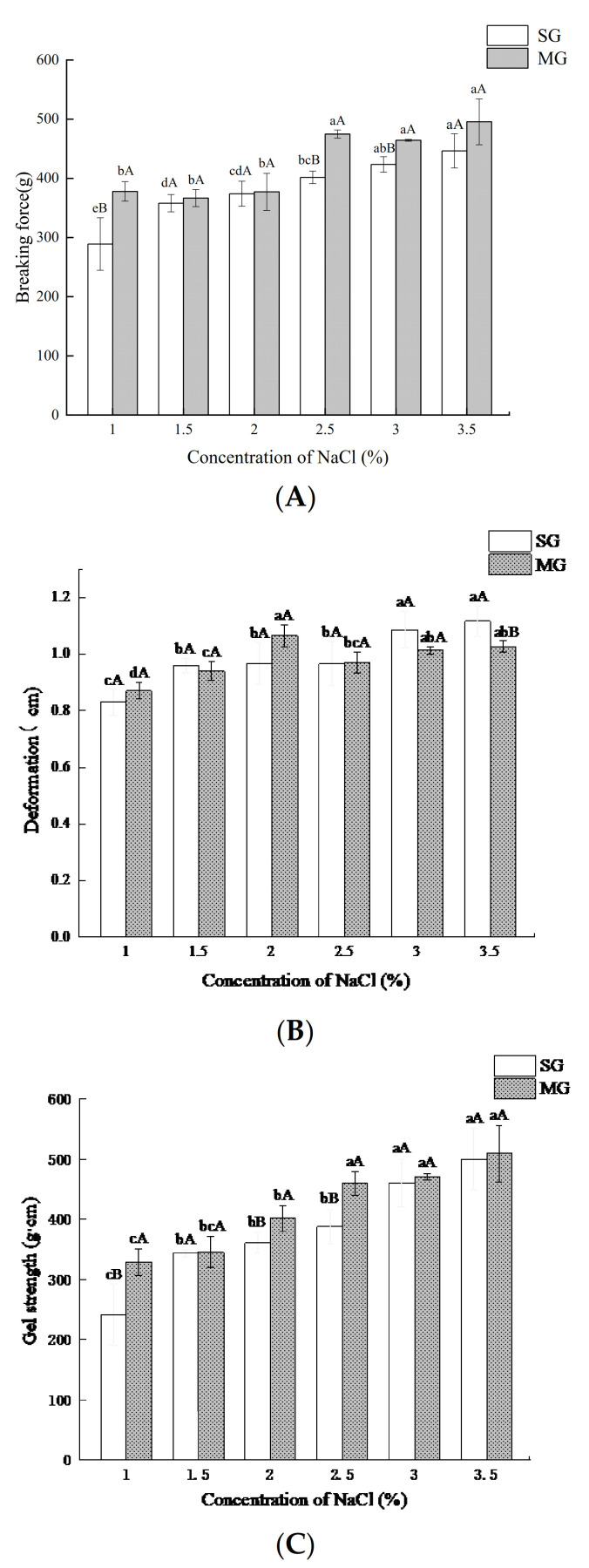
Effects of different NaCl concentrations on breaking force (**A**), deformation (**B**), and gel strength (**C**) of SG (surimi gels) and MG (mixed gels). Different lowercase letters show significant differences between the groups with different NaCl concentrations (*p* < 0.05). Different capital letters show significant differences (*p* < 0.05) between SG and MG.

We observed that NaCl addition significantly affected the SG (surimi gels) and MG (mixed gels)’s breaking force, deformation, and gel strength (Figure 1). The breaking force, deformation, and gel strength of SG gradually increased with the increase in salt concentration (1%, 1.5%, 2%, 2.5%, and 3%), but there was no significant difference (*p* > 0.05) in these properties of SG when the addition of NaCl was more than 3%. However, the breaking force increased in the MG while the deformation initially increased and then decreased with the increase in NaCl concentration. When the addition of NaCl exceeded 2.5%, no significant effect (*p* > 0.05) was observed on these MG properties. Salt is an indispensable component in surimi products, and the main function of salt is to extract the MP and induce the MP bonding to obtain the desired gel structure [21]. The textural properties in this study were inconsistent with those reported by Wang et al. [22], and they found that the myosin of silver carp spontaneously assembled in low concentration of NaCl environment (0.1 M), and a higher concentration of NaCl broke the ionic bond in myosin under electrostatic interaction and enhanced the interaction between protein and water. The addition of crabmeat may increase the hardness of the mixed gel system, resulting in an increase in the breaking force of MG. Simultaneously, excessive NaCl competed with protein for water, resulting in thick and nondense myosin filaments. Additionally, when the NaCl concentration was higher than 2.5%, it was difficult to interact between the crude myosin filament and crabmeat protein to make the gel network closer; therefore, the deformation decreased, and the breaking force did not change significantly. The results showed that 2.5% and 3% might be the best NaCl concentrations of MG and SG, respectively.

### 2.2. Water-Holding Capacity (WHC)

**Figure 2:**
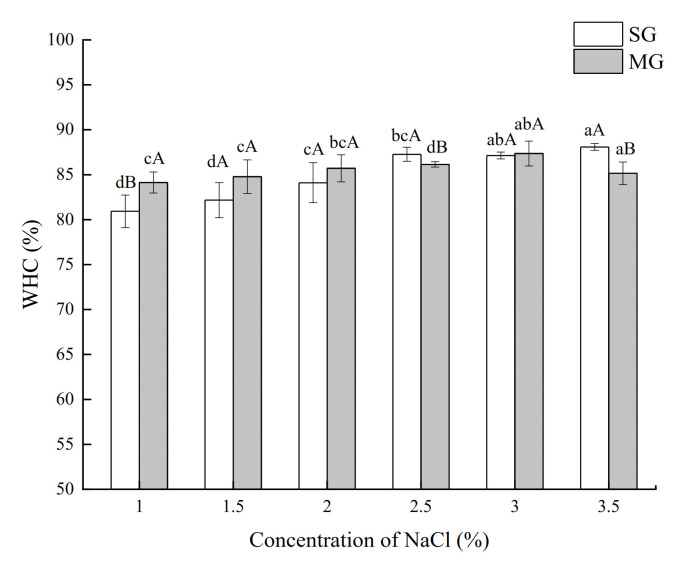
Effects of different NaCl concentrations on WHC of SG and MG. Different lowercase letters show significant differences between the groups with different NaCl concentrations (*p* < 0.05). Different capital letters show significant differences (*p* < 0.05) between SG and MG.

The WHC was an important indication representing the creation of surimi gel structure because water made up roughly 76% of surimi [23]. The addition of NaCl changed the WHC of both SG and MG. Overall, the WHC of both gels increased as the amount of NaCl added increased, indicating an improved structure of the gel network to store water (Figure 2). However, the WHC of MG increased with NaCl concentration and decreased (*p* > 0.05) when the NaCl addition was greater than 3%. The WHC of gels is determined by the type and number of protein–water interaction sites in the gels [24]. The force between the proteins or the interactions between the protein and water in the MG were impacted differently by the addition of NaCl than in the SG, possibly because the protein composition in crabmeat differed from that in surimi. Both the gel strength and WHC of SG are predominantly determined using salt-soluble proteins, and with the right salt concentration, MP would neither cause insufficient gel network formation due to low MP content nor prevent gel formation due to excessive MP aggregation before heating [25]. Higher levels of salt incorporation increased the WHC of farmed sea bass gels [26], and high concentrations (3%) of NaCl decreased the WHC of egg yolk gels [26,27].

### 2.3. Intrinsic Fluorescence Analysis

**Figure 3:**
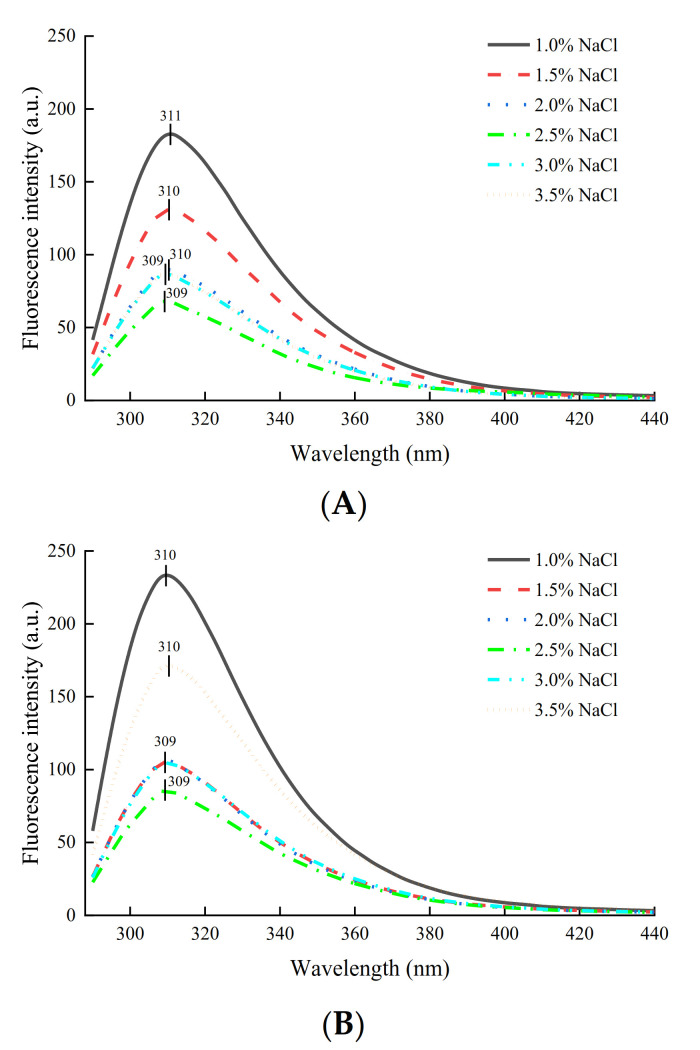
Effects of different NaCl concentrations on intrinsic fluorescence of SG (**A**) and MG (**B**).

Endogenous fluorescence is a sensitive indicator of protein unfolding and dynamics. The intrinsic fluorescence of proteins is primarily the contribution of natural chromophores, including tryptophan, tyrosine, and phenylalanine residues [28]. The fluorescence intensity of the SG gradually decreased with the increasing concentration of NaCl and then slightly increased (Figure 3). In the SG, the fluorescence intensity reached a minimum of 2.5% NaCl concentration, and the intrinsic fluorescence spectra of gel samples with 3% and 3.5% NaCl additions appeared to be similar. With the addition of NaCl, the fluorescence intensity of MG also initially decreased and then increased. When the fluorescence intensity of MG was the lowest, the concentration of NaCl was 2.5%, which was the same as the NaCl concentration at the lowest fluorescence intensity in SG. However, the fluorescence intensity of the MG sample added with 3.5% NaCl was significantly higher than that added with 2.5% NaCl. Tryptophan is wrapped in the inner core of folded natural protein molecules, showing high fluorescence intensity, and tryptophan residues are exposed to the solvent due to protein unfolding, resulting in reduced fluorescence intensity [29]. The gel formation of surimi was mainly due to the unfolding and aggregation of MP molecules. These results indicated that the aggregation of proteins in surimi increased with NaCl concentration, which gradually blocked the fluorescence signal of chromophores in proteins due to steric hindrance effects [30]. Moreover, it was reported that the increase in phosphorylation might make protein molecules easier to aggregate due to the increase in the electrostatic effect [31].

### 2.4. SDS–Polyacrylamide Gel Electrophoresis

**Figure 4:**
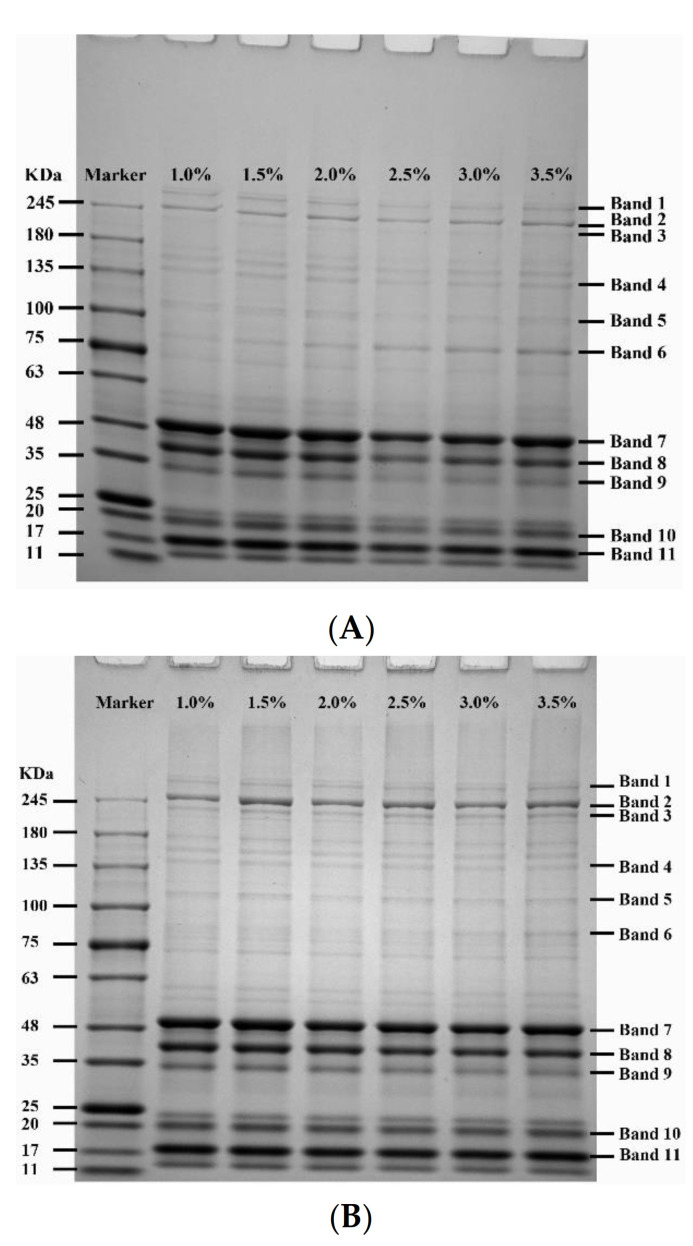

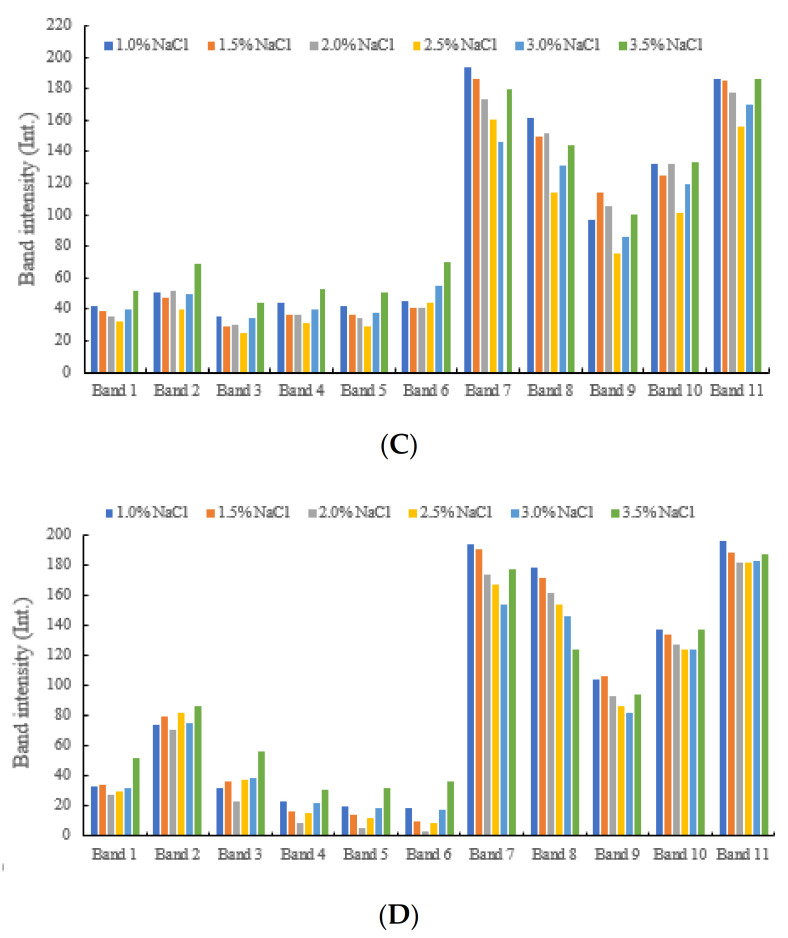
Effects of different NaCl concentrations on protein pattern of SG (**A**) and MG (**B**). (**C**): the intensity of bands in (**A**); (**D**): the intensity of bands in (**B**).

Protein patterns of SG and MG added with different levels of NaCl in the absence and presence of crabmeat are depicted in Figure 4. In the figure, the intensity of the 11 bands initially decreased and then increased with NaCl addition, following a similar trend. Band 3 was MHC (220 KDa), and band 7 was AC (49 KDa). It was reported that the proteins in surimi paste mainly included MHC and AC [32]. During the transformation of surimi paste into a gel, MHC molecules interact to form complex aggregates, which finally form a network structure, and thus, MHC bands gradually fade. However, AC was the dominant protein in the gel compared to the MHC, indicating that actin has strong resistance to protein degradation or less participation in polymerization during the gelation process [33]. The interaction between protein molecules was not enough at a low-salt content (1%, 1.5%, 2%), and high-molecular-weight proteins were unable to aggregate and were easy to degrade into small molecular weight proteins, so the intensity of protein bands was increased. In the protein pattern of SG and MG, the intensity of most protein bands was low when the salt content was 2.5% and 3%, which suggested that there were larger aggregates formed by protein interaction in surimi at higher salt concentrations, resulting in a more compact three-dimensional network structure. However, the interaction between small molecular proteins might be further enhanced to form small aggregates that easily degrade when the salt concentrations are too high (3.5%) [22]. From the result, the decrease in the intensity of the protein bands was coincidental with the increase in the breaking force and deformation (Figure 1). In MG, bands 4, 5, and 6 were lower, while bands 2, 7, 8, 10, and 11 each showed a higher intensity than that in the SG, which might reveal that the interactions between different proteins in surimi and crabmeat were different.

### 2.5. Determination of the Degree of Phosphorylation

**Figure 5:**
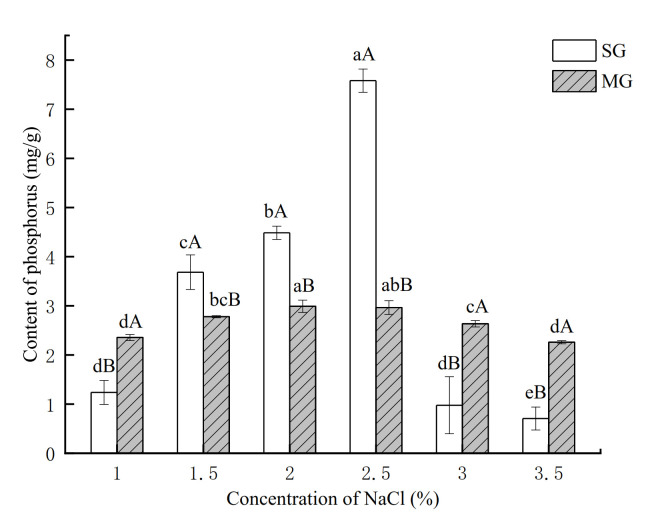
Effects of different NaCl concentrations on the degree of phosphorylation of SG and MG. Different lowercase letters show significant differences between the groups with different NaCl concentrations (*p* < 0.05). Different capital letters show significant differences (*p* < 0.05) between SG and MG.

The phosphorylation degree of proteins in SG and MG with different salt additions is shown in Figure 5. Compared with other groups with different additions of NaCl, the phosphorus content attached to proteins in SG with 2.5% NaCl increased significantly (*p* < 0.05) and the phosphorus content of MG added with 2% NaCl increased significantly (*p* < 0.05), but there was no difference between the 2% and 2.5% group of MG, suggesting that the addition of NaCl had an obvious effect on protein phosphorylation during the formation of surimi products. Our findings were consistent with the reduced phosphorylation level of myofibrillar protein in salted meat reported by Zhang et al. [20], in which phosphorylated MP, such as actin-binding protein, exhibited stronger resistance to calpain-induced degradation than those that were not phosphorylated. It was discovered that the phosphorylation of the myosin regulatory light chain (MRLC) in rabbit muscle inhibited actomyosin dissociation during incubation and that the phosphorylation of MRLC at Ser17 promoted the formation of ionic bonds, hydrogen bonds, and hydrophobic interactions between myosin and actin [34]. The reduction in AC band intensity in both gels with 2% and 2.5% NaCl additions was observed in the result of the protein pattern (Figure 4), which might be related to the inhibition of actin dissociation, the reduction in degradation, and the promotion of cross-linking between MHC by phosphorylation level, thus improving the gel properties of surimi and crabmeat.

### 2.6. Western Blotting Analysis

**Figure 6:**
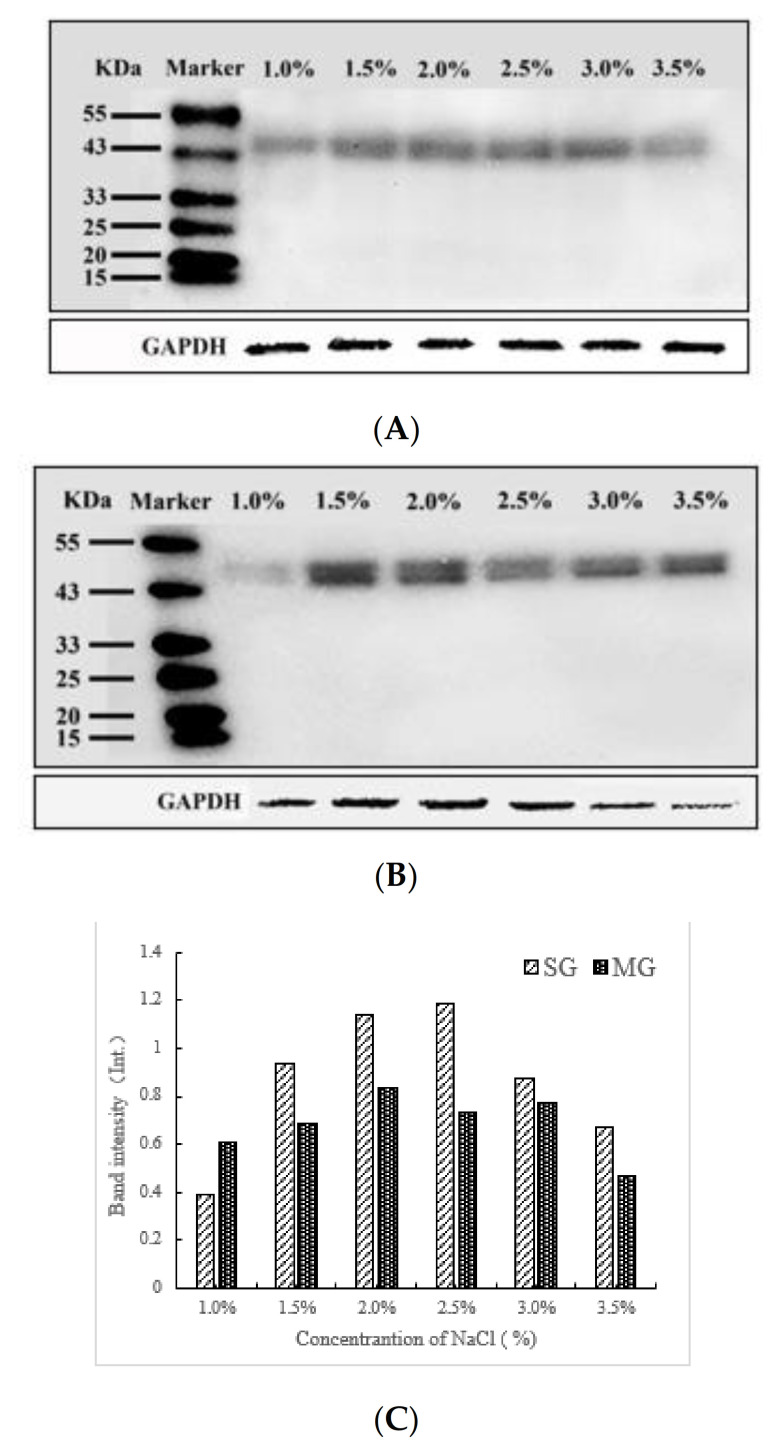
Determination of Western blot of SG (**A**) and MG (**B**) with different NaCl concentrations. (**C**): the intensity of bands in (**A**,**B**).

After membrane transfer, the proteins in the SDS–PAGE gel were analyzed using a Western blot. No protein bands were observed except the AC band due to the species and abundance of phosphorylated proteins. The intensity of AC protein bands was quantified using optical density measurement to compare the differences between samples with different NaCl additions. As shown in Figure 6, the protein phosphorylation of both gel samples increased and reached the maximum at 2% and 2.5% NaCl with the increase in NaCl concentration, and then gradually decreased. This was consistent with the changing trend in the determination results of phosphorus content attached to a protein (Figure 5). However, the amplitude of variation was different, indicating that the degree of phosphoprotein participating in the aggregation of macromolecular proteins that SDS–PAGE did not separate was different. One reason could be that phosphorylation increased the negative charge of the gel matrix and promoted the thermal polymerization of surimi pastes or mixed pastes during heating [30], resulting in a sharp decrease in the intensity of phosphorylated protein bands in SG with 2.5% NaCl in Western blot analysis compared with the result of the determination of the degree of phosphorylation (Section 2.5). Furthermore, compared with the results of Section 2.5, it can be seen that when the NaCl concentrations were 3% and 3.5%, the phosphorylation degree of MG was higher than that of SG (Figure 5), while the phosphorylation protein band intensity of MG was lower than that of SG in Figure 6. This suggested that the incorporation of crabmeat had different effects on protein phosphorylation of SG at high NaCl concentrations. It was hypothesized that the phosphorylated proteins of MG in an environment with a high concentration of NaCl tend to aggregate with each other to form small aggregates that are easily decomposed, based on the results of the enhanced fluorescence intensity of the MG with 3.5% NaCl added in the intrinsic fluorescence analysis (Figure 3) [35].

## 3. Conclusions

Our results showed that a certain concentration of salt (2–2.5%) was associated with an increase in protein phosphorylation of SG and MG, accompanied by an increase in breaking force, deformation, gel strength, and WHC of gels. Phosphorylation promoted the interaction between proteins to form macromolecular protein polymers, resulting in decreased protein band intensity in the SDS–PAGE protein pattern and intrinsic fluorescence intensity. Furthermore, the effects of salt on the gelling properties and protein phosphorylation of MG were different from those of pure SG. Our study provided a new direction for producing low-salt surimi products and the development of low-value precocious crabs. However, the effect of protein phosphorylation on protein conformation and the deeper interaction mechanism of proteins in surimi products are required for further investigation.

## 4. Materials and Methods

### 4.1. Materials

Grade AAA-frozen silver carp surimi containing 6% sucrose in 20 kg blocks was generously provided by Jingli Aquatic Food Co., Ltd. (Honghu, Hubei, China). It was stored at −20 °C for no longer than two months. The crabmeat was obtained from precocious Chinese mitten crab purchased from Fuen Food Technology Co., Ltd. (Maanshan, Anhui, China). The crabmeat was handpicked from each section of the steamed crab and stored at −20 °C.

Phospho-threonine/tyrosine antibody and anti-rabbit IgG (HRP-linked) antibody were purchased from Cell Signaling Technology Co., Ltd. (Danvers, MA, USA). Anti-GAPDH antibody was purchased from Sangon Biotech Co., Ltd. (Shanghai, China). Other chemicals used in the study were purchased from Meifeng Technology Co., Ltd. (Hefei, Anhui, China) or Solarbio Science & Technology Co., Ltd. (Beijing, China). Solarbio Science & Technology Co., Ltd. (Beijing, China) supplied the SDS lysis solution and ECL plus super-sensitive luminescent liquid. All the reagents used were of analytical grade.

### 4.2. Preparation of Surimi and Crabmeat Mixed Gels

Surimi gels were prepared according to the method described by Liang et al. [11]. Appropriate amounts of frozen surimi and crabmeat were removed from the freezer and thawed overnight at 4 °C. About 90% frozen surimi and 10% crabmeat (based on the total mass of surimi and crabmeat) were cut into small pieces and chopped into surimi and crabmeat mixed pastes at 1500 rpm for 1 min in a chopper (Guangzhou Xuzhong Food Machinery Co., Ltd., Guangzhou, China). Then, different additive amounts of solid NaCl were added to the mixed pastes (1%, 1.5%, 2%, 2.5%, 3%, and 3.5% based on the total mass of the mixtures), and the mixtures were further chopped at 1800 rpm for 2 min. Different mixed pastes were added with cold distilled water to adjust the same moisture content of 76%. The prepared mixed pastes were stuffed into polyvinylidene chloride casings (30 mm in diameter) with both ends sealed tightly using U-shaped aluminum wire clips (Ruian Special Feng Machinery Plant, Zhejiang, China). The samples were then incubated at 40 °C for 1 h, followed by a heating period at 90 °C for 30 min, to form surimi gels. After heating, the surimi and crabmeat sausages were cooled in ice water immediately for 15 min and stored overnight at 4 °C to determine the properties. In this study, the gels containing different amounts of salt were divided into two groups: surimi gels (SG; without crabmeat) and mixed gels (MG; with 10% crabmeat).

### 4.3. Puncture Test

Using a TA-XT texture analyzer, the breaking force (g) and penetration distance (cm) of the SG and MG samples were analyzed (Stable Micro System, Surrey, UK). The samples were equilibrated at room temperature (24–26 °C) for 1 h and then cut into cylinders (30 mm diameter and 25 mm height). The specific settings and parameters were as follows: surimi penetration mode, P/5S spherical probe (diameter = 5 mm), pre-compression speed = 1.0 mm/s, down-compression speed = 1.5 mm/s, test speed = 1.0 mm/s, recovery speed = 10.0 mm/s, pressing distance = 15 mm, trigger type = auto (force), and sensing force = 5.0 g. Measurements were performed in sextuplicate for each sample, and averages were calculated from the final data after deleting the maximum and minimum values. The gel strength was calculated using the product of breaking force and deformation.

### 4.4. Water-Holding Capacity (WHC)

The analysis of WHC was performed using a centrifuge method based on the method described by Liang et al. [36], with slight modifications. All the 2-mm-thick gel samples (1–2 g) were accurately weighed (*W*_1_) and cut into two equal portions, which were wrapped in two layers of filter paper and transferred into 50 mL centrifuge tubes. Each sample was centrifuged for 10 min at 8000 rpm at room temperature (24–26 °C) (Tianmei biochemical equipment Engineering Co., Ltd., Shanghai, China). After centrifugation, the samples were weighed again (*W*_2_). Each experiment was performed three times. The WHC of each sample was calculated according to Equation (1):

(1) $$\mathrm{WHC}\left(\%\right)=\frac{W_{2}}{W_{1}}\times 100\%$$

### 4.5. Intrinsic Fluorescence Analysis

The intrinsic fluorescence emission spectra of SG and MG were determined at room temperature (24–26 °C) using a FLS980 fluorescence spectrophotometer (Edinburgh Instruments Co., Ltd., Edinburgh, UK). The gel sample was ground into a powder after vacuum freeze-drying, and the powder was dispersed in 0.01 M phosphate buffer (pH 7.5) to generate a gel protein solution, according to Wang, Yang, Fan, Zhang, and Chen [30]. The protein suspensions were diluted to a final concentration of 0.2 mg/mL and stimulated at 290 nm (slit width 3 nm). The emission spectra were acquired at a scanning speed of 5 nm/s between 300 and 450 nm, and background spectra were recorded and removed from the treated samples under the same conditions.

### 4.6. Determination of the Degree of Phosphorylation

The phosphorus content of the samples indicated the degree of phosphorylation. After some modifications, the phosphorylation degree was determined using the methods of Ai and Jiang [37] and Hu, Qiu, Sun, Xiong, and Ogra [38]. A kit was used to determine the total phosphorus and inorganic phosphorus content (BC2850 and BC2845, Solarbio Science & Technology Co., Ltd. Beijing, China). Briefly, freeze-dried SG and MG were crushed into powders and then 0.01 g of sample powder was digested in 1 mL of concentrated sulfuric acid and heated in boiling water for 10 min until it became black or brown. Reagent A1 (from the BC2850 kit) was added to the solution after it had cooled and was thoroughly mixed. Then, the solution was kept in a boiling water bath until it was transparent.

Phosphorus in the digested solution was the total phosphorus (*P_T_*) of the surimi and crabmeat MG. To determine inorganic phosphorus (*P_I_*), 1 mL Reagent A_2_ (from BC2845 kit) was added to 0.05 g powder sample, and the mixture was homogenized before being centrifuged for 10 min at 10,000 rpm (on ice, 4 °C). The *P_I_* was determined by measuring the amount of phosphorus in the supernatant. The phosphorus concentration was determined using the molybdenum blue colorimetry: Reagent C (containing molybdate, from BC2845 or BC2850 kit) was added to the solution and incubated for 10 min in a 40 °C water bath. Then, the solution was cooled at room temperature for 10 min and the absorbance was read in a microplate reader (EPOCH, Bio Tek Instruments Inc., Winooski, Vermont (VT), USA) at 660 nm. The differences between *P_T_* and *P_I_* were used to estimate the amount of phosphorus bound to proteins. The phosphorus was calculated according to Equation (2):

(2) $$P=\frac{P_{T}-P_{I}}{m}$$

where P represents the phosphorus content bound to protein (mg/g), *P_T_* is the total phosphorus content (mg), *P_I_* is the inorganic phosphorus content (mg), and *m* is the weight of the detected sample (g).

### 4.7. SDS–Polyacrylamide Gel Electrophoresis

The protein of samples was extracted by the Kudre, Benjakul, and Kishimura [39] method, with some modifications. Freeze-dried SG and MG were crushed into powders. Then, 0.02 g of sample powder was diluted in 1 mL SDS Lysis Buffer (R0070, Solarbio Science & Technology Co., Ltd., Beijing, China), which contained 50 mM tris (pH 8.1), 1% SDS, sodium pyrophosphate, sodium orthovanadate, EDTA (Ethylene Diamine Tetraacetic Acid), and leupeptin. Then, 10 μL 1 mmol/L PMSF (phenylmethylsulfonyl fluoride) was added, and the mixture was centrifuged at 12,000 rpm for 8 min after being fully homogenized on ice (4 °C). After 10 min in a boiling water bath, three times the volume of the supernatant was added to the loading buffer, and the protein solution was stored at −20 °C before analysis. Protein patterns of the gels were analyzed by SDS–polyacrylamide gel electrophoresis (SDS–PAGE) according to the modified method of Laemmli [40] using a 4% stacking gel and a 15% separating gel (Sangon Biotech Co., Ltd. Shanghai, China). The protein solution was centrifuged at 10,000 rpm for 5 min, and then 10 μL samples were loaded in the electrophoresis gel. Electrophoresis was performed at a voltage of 80 V. After the tracking blue dye migrated at the end of the gels, the gels were removed and stained with a dye solution containing 0.1% (*w*/*v*) Coomassie Brilliant Blue R-250 in 45% (*v*/*v*) methanol, 10% (*v*/*v*) acetic acid, and double-distilled water. After 25 min of dyeing, the gels were destained with a solution of 10% (*v*/*v*) methanol and 10% (*v*/*v*) acetic acid until the gel background was clear enough to be photographed.

### 4.8. Western BlotAnalysis

To evaluate the phosphorylated proteins in all samples, Western blotting was used, with minor changes, as described by Towbin, Staehelin, and Gordon [41] and Lv et al. [42]. The same conditions were used for SDS–PAGE as in Section 4.7. Following SDS–PAGE, the 15% acrylamide gel protein was transferred onto a polyvinylidene fluoride (PVDF) membrane in a transfer buffer. Then, 5% bovine serum albumin was used to block nonspecific protein sites at room temperature (24–26 °C) for 1 h. After blocking, the membrane was incubated at 4 °C overnight with corresponding diluted primary antibody, and the unbound antibody was removed by washing the membrane with TBST (20 mM Tris-HCl containing Tween-20, pH 7.5). Then, the membrane was incubated with horseradish peroxidase-conjugated secondary antibody for 1 h, followed by extensive washes with TBST. The ECL then detected the result and photographed it using a FluorChem E System (Protein Simple, San Jose, CA, USA).

### 4.9. Statistical Analysis

Using a 95% confidence range, analysis of variance was used to determine the significance of the differences between the experimental data acquired from the samples. The Statistical Package for the Social Sciences was used for statistical analysis (SPSS 25.0, IBM, SPSS Inc., Chicago, IL, USA). The data were presented as mean values with standard deviation.

## Data Availability

Not applicable.
